# Neighborhood socioeconomic characteristics and statin medication in patients with myocardial infarction: a Swedish nationwide follow-up study

**DOI:** 10.1186/s12872-016-0319-y

**Published:** 2016-07-08

**Authors:** Per-Ola Forsberg, Xinjun Li, Kristina Sundquist

**Affiliations:** Center for Primary Health Care Research, Clinical Research Centre (CRC), Lund University, building 28, floor 11, Jan Waldenströms gata 35, SE-205 02 Malmö, Sweden

**Keywords:** Myocardial infarction, Neighborhood deprivation, Socioeconomic status, Statins

## Abstract

**Background:**

Coronary heart disease (CHD) and myocardial infarction (MI) are associated with neighborhood-level socioeconomic status (SES). Statins are important drugs for secondary prevention of MI. However, no study has determined whether neighborhood-level SES is associated with statin medication in MI patients. We aimed to determine whether there is a difference in statin medication rate in MI patients across different levels of neighborhood SES.

**Methods:**

All patients in Sweden, diagnosed with incident MI from January 1st, 2000 until December 31^st^ 2010, were followed (*n* = 116,840). Of these, 89.7 % received statin medication. Data were analyzed by multilevel logistic regression, with individual-level characteristics (age, marital status, family income, educational attainment, country of origin, urban/rural status and comorbidities/chronic conditions related to MI) as covariates.

**Results:**

Low neighborhood-level SES was significantly associated with low statin medication rate (Odds Ratio 0.80). In the full model, which took into account individual-level socioeconomic characteristics and MI comorbidities, the odds no longer remained significant.

**Conclusions:**

Individual-level approaches may be most important in health care policies regarding statin medication in MI patients.

**Electronic supplementary material:**

The online version of this article (doi:10.1186/s12872-016-0319-y) contains supplementary material, which is available to authorized users.

## Background

Coronary heart disease (CHD) and in particular myocardial infarction (MI) is a major cause of morbidity, mortality and health expense worldwide [1]. Up to 70 % of CHD incidence can be explained by individual-level sociodemographic characteristics (age, sex, socioeconomic status (SES)); health behaviors (smoking, physical inactivity, poor diet); and metabolic risk factors (hypertension, diabetes, hypercholesterolemia) [2]. Today, CHD prevention includes both primary and secondary prevention with lifestyle changes as well as medical treatment of risk factors, primarily hypertension, diabetes, and hypercholesterolemia. HMG CoA-reductase inhibitors, or statins, are a class of cholesterol lowering drugs that are widely used in this context, in particular for secondary prevention of MI [3]. Statins reduce both mortality and morbidity for CHD patients [4].

Although individual-level sociodemographic characteristics, such as low individual SES, are important risk factors for CHD incidence, recent work has also shown that low neighborhood SES is associated with an increased risk of CHD, after adjustment for individual SES [5–9].

However, no study has examined whether neighborhood SES is associated with statin medication rates, after taking individual socioeconomic factors into account. Such studies are important because if there are differences in statin medication by certain neighborhood-level characteristics, interventions will need to target such neighborhoods.

Sweden is a suitable setting for such a study because it is a country which has universal health coverage for all residents and good availability of health-related resources [10]. It is therefore possible to examine whether statin medication patterns differ, by both individual and neighborhood level SES, in a country that has almost equal availability of health care.

The aim of this study is to determine whether the statin medication rates for MI patients differ across neighborhoods with different levels of neighborhood SES, after taking individual factors into account.

## Methods

All data were linked by the personal Swedish identification number, which was replaced by a serial number in the dataset in order to maintain anonymity for all individuals. Health care data were retrieved from nationwide Swedish in-patient as well as out-patient specialist care; the latter in order to increase the number of cases as well as generalizability of findings. The Swedish Hospital Discharge Register contains nationwide hospital main and secondary discharge diagnoses encoded in the International Classification of Diseases (ICD) format. The tenth revision (ICD-10) has been in use since 1997 [11]. The diagnoses in the Hospital Discharge Register have a positive predictive value between 85 and 95 %, and the validity of many cardiovascular diseases are even higher; e.g., MI, angina pectoris and atrial fibrillation have a positive predictive value of > 95 % [11–13]. Since 2000, the proportion of missing primary diagnoses has been only 0.5–0.9 % [11].

Inclusion criteria was MI registered as the main diagnosis in the Hospital Discharge Register or Outpatient Register within the study interval (2000 to 2010) using ICD-10 codes I21, I22, and I23. Only patients who previously had no CHD were included. This was achieved by excluding patients with a recorded main or secondary diagnosis of CHD (ICD-10 I20-I25) during a 3-year period, i.e., from 1997 to 1999, before study start. Patients, who died between 1 January 2000 and 30 June 2005, were excluded as medication data was not available for this time period. Also, all patients who died within one month of MI diagnosis were excluded, as we assumed that some of them might not have had a chance to pick up the medication. Patients above 80 years of age were excluded as the proportion of patients aged 80+ with statin medication was low.

### Outcome (dependent) variable

The outcome variable of medication of statins was defined according to the ATC code C10AA for the Medication Register.

The statins (HMG CoA reductase inhibitors, code C10AA) included were:

- C10AA01 Simvastatin
- C10AA02 Lovastatin
- C10AA03 Pravastatin
- C10AA04 Fluvastatin
- C10AA05 Atorvastatin
- C10AA06 Cerivastatin
- C10AA07 Rosuvastatin
- C10AA08 Pitavastatin

### Individual-level variables

The individual-level variables used in models were sex, age at the start of the study, marital status, family income, education level, country of origin, urban/rural status, and chronic conditions related to MI (comorbidities) [14–16].

*Sex*: Male or female.

*Age* was assessed at start of follow-up. Age was used as a continuous variable in the models.

*Marital status*: Individuals were classified as married/cohabitating or widowed/divorced/never married.

*Family income by quartile*: Information on family income in 2001 came from the Total Population Register, provided by Statistics Sweden. Income was categorized into quartiles: low income, middle-low income, middle-high income, and high income. The income was divided by the number of people in the family. A weighted system was also used; small children were given lower weights than adolescents since the costs of living for a small child are lower than those for an adolescent.

*Education level* was classified as completion of compulsory school or less (≤9 years), vocational high school or some theoretical high school (10–12 years), and completed theoretical high school and/or college (>12 years).

*Country of origin*: Born in: 1) Sweden (reference), 2) Finland, 3) Western countries, 4) Eastern European countries, 5) Middle Eastern countries, or 6) other countries. *Urban/rural status*: Residence in major cities (Stockholm, Gothenburg or Malmö), southern or northern Sweden.

*Chronic conditions related to MI--comorbidities* were defined as the first diagnosis (main or additional diagnosis) ten years before the study and during the follow-up period of: 1) chronic lower respiratory diseases (ICD-9: 490–496; ICD-10: J40-J49), 2) alcoholism and alcohol-related liver disease (ICD-9: 291, 303, and 571; ICD-10: F10 and K70), 3) hypertension (ICD-9: 401–405; ICD-10: I10-I19), and 4) diabetes mellitus (ICD-9: 250; ICD-10: E10-E14).

### Neighborhood-level socioeconomic status (SES)

The home addresses of all Swedish individuals have been geocoded to small geographical units that have boundaries defined by homogeneous types of buildings. These neighborhood areas, developed by Statistics Sweden, are called small areas for market statistics (SAMS) and have an average of 1000 people each. SAMS were used as proxies for neighborhoods, as in previous research [7, 17]. A summary index was calculated to characterize neighborhood-level SES [5]. The neighborhood index was based on information on men and women aged 20–64, who lived in the neighborhood, because people in this age group are the most socioeconomically active. In other words, as a population group they have a stronger impact on the socioeconomic structure of the neighborhood than children, younger men/women and retirees. The neighborhood index was based on four items: low education level (<10 years of formal education), low income (income from all sources, including that from interest and dividends, defined as less than 50 % of the median individual income), unemployment (excluding full-time students, those completing compulsory military service, and early retirees) and receipt of social welfare. The index was categorized into the following three groups: low neighborhood SES (more than 1 SD below the mean), middle neighborhood SES (within 1 SD of the mean), and high neighborhood SES (more than 1 SD above the mean) [5]. The neighborhood SES index in the year 2000 was used in the models.

### Statistical methods

Multilevel (hierarchical) logistic regression models were used to estimate odds ratios (OR) for statin medication rates for different levels of neighborhood SES. The analyses were performed using MLwiN version 2.27. The first model only included neighborhood-level SES to determine the crude odds of statin medication by level of neighborhood SES (Model 1). The second model also included the individual-level characteristics age and sex (Model 2). The third model added family income, marital status, country of origin, educational attainment and urban/rural status (Model 3). Last, a full model was created which also included hospitalization due to chronic lower respiratory disease, alcoholism and related liver disease, type 2 diabetes mellitus or hypertension (Model 4, not shown in tables).

#### Random effects

The between-neighborhood variance was estimated with a random intercept. It was regarded to be significant if it was more than 1.96 times the size of the standard error, in accordance with the precedent set in previous studies [18–20].

We computed the intraclass correlation (ICC)—that is, the intra-neighborhood correlation—in order to estimate to what extent the individual chance of statin medication for individuals, within the same SAMS, was similar compared with individuals in other SAMS areas. The ICC expresses the proportion of the total variance that is at the neighborhood level. The ICC in multilevel logistic regression can be estimated by different procedures. We applied the latent variable method [21] as exemplified by:

$$ ICC=\frac{V_n}{V_n+{\pi}^2/3} $$

where Vn is the variance between neighborhoods and π^2^/3 is the variance between individuals.

The proportion of the second level variance explained by the different variables was calculated as:

$$ {V}_{EXPLAINED}=\frac{V_0-{V}_1}{V_0}\times 100, $$

where Vo is the age adjusted variance in the initial model and V_1_ is the second level variance in the different models.

First order interactions between neighborhood deprivation and individual-level characteristics for statin medication in MI patients were also analyzed.

For comparison, we also calculated logistic regression models using the SAS statistical package (version 9.3; SAS Institute, Cary, NC, USA).

## Results

Table 1 shows the distribution of the study population and number of patients receiving statins by neighborhood-level SES. For statin medication, the number (No) of patients receiving statins as well as the share of patients on statin medication within each patient group (%) is presented. Of the 116,840 patients with MI included in this study, 104,766 (89.7 %) received statins during the study period. Of the total population, 19 %, 62 %, and 19 % lived in high, middle, and low SES neighborhoods, respectively. Age-standardized statin medication rates was 90.6 % in neighborhoods with high SES; 89.7 % in neighborhoods with middle SES; and 88.6 % in neighborhoods with low SES. This slight difference in statin medication rate by neighborhood-level SES was observed across all individual-level variables, as shown in Table 1.

**Table 1:** Distribution of study population (patients with myocardial infarction, MI), number of statin medication events, and age-standardized rates by neighborhood socioeconomic status (SES)

	Population	Distribution	Statin medication		Neighborhood SES		
		(%)	No.	%	High	Middle	Low
Total population (%)	116,840				22,172 (19 %)	72,655 (62 %)	22,013 (19 %)
Statin medication			104,766	89.7	90.6	89.7	88.6
Sex							
Men	80,351	68.8	73,223	91.1	91.8	90.6	89.0
Women	36,489	31.2	31,543	86.4	87.5	87.6	87.4
Age (years)							
< 50	18,623	15.9	17,185	92.3	92.1	92.5	91.7
50–59	34,009	29.1	32,147	94.5	95.1	94.6	93.6
60–69	40,034	34.3	36,786	91.9	92.3	92.1	90.8
70–79	24,174	20.7	18,648	77.1	78.6	77.4	74.8
Educational attainment							
≤ 9	55,061	47.1	47,813	86.8	89.5	88.9	87.9
10–12	31,699	27.1	29,147	91.9	91.2	91.2	89.5
> 12	30,080	25.7	27,806	92.4	92.2	91.3	89.9
Marital status							
Married/cohabiting	71,108	60.9	64,923	91.3	91.6	91.4	91.0
Never married, widowed, or divorced	45,732	39.1	39,843	87.1	88.4	87.1	85.9
Family income							
Low income	29,298	25.1	25,201	86.0	88.1	87.7	87.5
Middle–low income	29,175	25.0	25,538	87.5	89.1	89.1	87.2
Middle–high income	29,202	25.0	26,812	91.8	90.8	91.0	90.5
High income	29,165	25.0	27,215	93.3	92.1	91.6	91.7
Country of origin							
Sweden	99,947	85.5	89,581	89.6	90.6	89.8	88.5
Finland	5113	4.4	4568	89.3	90.4	88.2	87.0
Western countries	1379	1.2	1235	89.6	91.2	88.9	90.6
Eastern European countries	2500	2.1	2310	92.4	90.7	90.6	89.8
Middle Eastern countries	2469	2.1	2287	92.6	88.1	88.9	90.1
Others	5432	4.6	4785	88.1	89.3	88.4	86.5
Urban/rural status							
Large cities	52,572	45.0	46,829	89.1	90.1	89.0	87.6
Southern Sweden	41,907	35.9	38,125	91.0	92.2	90.9	90.4
Northern Sweden	22,361	19.1	19,812	88.6	89.5	88.7	87.7

Table 2 shows the age-adjusted ORs for each covariate. Patients with low family income had significantly lower odds of statin medication (OR 0.49) compared to patients with high family income. Low educational attainment was also associated with lower odds of statin medication (OR 0.71) compared to patients with high educational attainment.

**Table 2:** Odds ratios (OR) and 95 % confidence intervals (CI) for statin medication in patients with myocardial infarction; Results of multi-level logistic regression models for each variable, adjusted for age

	OR	95 % CI		Variance (S.E.)	*Explained variance (%)*	*Intra class correlation*
Neighborhood-level Socioeconomic status (SES, ref. High)				0.068 (0.011)	22	0.020
Middle	0.93	0.88	0.98			
Low	0.79	0.74	0.84			
Sex (ref. Female)	1.39	1.34	1.45	0.072 (0.011)	17	0.021
Family income (ref. High income)				0.077 (0.011)	11	0.023
Low income	0.49	0.47	0.52			
Middle-low income	0.59	0.56	0.63			
Milddle-high income	0.84	0.79	0.89			
Marital status (ref. Married/co-habiting)				0.060 (0.011)	31	0.018
Never married, widowed, or divorced	0.59	0.57	0.62			
Country of origin (ref. Born in Sweden)				0.075 (0.011)	14	0.022
Finland	0.90	0.82	0.99			
Western countries	1.06	0.89	1.26			
Eastern European countries	1.10	0.94	1.28			
Middle eastern countries	0.99	0.84	1.16			
Others	0.80	0.74	0.88			
Educational attainment (ref. > 12 years)				0.075 (0.011)	14	0.022
≤ 9 years	0.71	0.67	0.74			
10–12 years	0.92	0.87	0.98			
Urban/rural status (ref. large cities)				0.059 (0.011)	32	0.018
Southern Sweden	1.28	1.22	1.34			
Northern Sweden	0.98	0.93	1.03			

Table 3 shows the results of the different multilevel logistic regression models. In the crude model (Model 1), the odds of statin medication were lower for patients living in neighborhoods with low SES. The OR for statin medication in patients with MI living in neighborhoods with low compared to high neighborhood-level SES was 0.80 (95 % confidence interval (CI) 0.75–0.86). In Model 2, neighborhood-level SES remained significantly associated with statin medication after adjustment for age and sex (OR 0.80, 95 % CI 0.75–0.86).

**Table 3:** Odds ratios (OR) and 95 % confidence intervals (CI) for statin medication in patients with myocardial infarction; Results of multi-level logistic regression models

	Model 1			Model 2			Model 3			
	OR	95 % CI		OR	95 % CI		OR	95 % CI		
Neighborhood-level socioeconomic status (SES, ref. High)										
Middle	0.90	0.86	0.95	0.94	0.89	0.99	1.07	1.01	1.13	0.016
Low	0.80	0.75	0.86	0.80	0.75	0.86	1.03	0.96	1.10	0.368
Age (years)				0.95	0.95	0.95	0.96	0.96	0.96	<0.001
Sex (ref. Female)				1.39	1.33	1.44	1.23	1.19	1.29	<0.001
Family income (ref. High income)										
Low income							0.49	0.46	0.52	<0.001
Middle-low income							0.62	0.58	0.65	<0.001
Milddle-high income							0.83	0.78	0.89	<0.001
Marital status (ref. Married/co-habiting)										
Never married, widowed, or divorced							0.61	0.58	0.63	<0.001
Country of origin (ref. Born in Sweden)										
Finland							1.02	0.93	1.12	0.689
Western countries							1.07	0.89	1.27	0.484
Eastern European countries							1.33	1.14	1.55	<0.001
Middle eastern countries							1.33	1.13	1.56	<0.001
Others							0.92	0.84	1.00	0.046
Educational attainment (ref. > 12 years)										
≤ 9 years							0.89	0.84	0.94	<0.001
10–12 years							1.13	1.06	1.21	<0.001
Urban/rural status (ref. large cities)										
Southern Sweden							1.30	1.24	1.37	<0.001
Northern Sweden							1.02	0.97	1.08	0.424
Variance (S.E.)	0.082 (0.011)			0.067 (0.011)			0.053 (0.011)			
Explained variance (%)	6			23			39			
Intra class correlation	0.024			0.020			0.016			

Model 1. Crude model; Model 2: Adjusted for age and sex; Model 3: Adjusted for age, sex, and individual socioeconomic variables

However, in the third model, after adding individual-level sociodemographic variables, the ORs no longer remained significant. The fourth model also included the variables for comorbidities, which did not change the ORs further, and the results are therefore not shown.

The between-neighborhood variance was significant in all models. The explained variance was 6 % in Model 1, indicating that the neighborhood variable explained only a small proportion of the total variance, and increased to 23 % in Model 2 and 39 % in Model 3.

The ICC was low, varying between 1.6 and 2.4 % in the different models, indicating that the clustering within neighborhoods was low.

Analyzing first order interactions between neighborhood SES and individual-level characteristics for statin medication in MI patients showed significant interactions between neighborhood level SES and education level only. The results of this analysis are shown in Additional file 1: Table S1.

## Discussion

The present study shows that MI patients living in low SES neighborhoods had 20 % lower odds of statin medication compared to those living in middle and high SES neighborhoods. However, the odds no longer remained significant after adjusting for the individual-level sociodemographic characteristics and comorbidities. To the best of our knowledge, the potential influence of neighborhood SES on statin use in MI patients has not been examined previously.

Previous studies have shown that statin medication rates in CHD patients may vary due to individual-level factors such as the patients’ age and comorbidities. In addition, low individual-level SES may affect medication patterns negatively [22–25] and it has been shown that when a larger proportion of the medication costs are required from the patients, statin utilization decreases, in particular for less regularly compliant patients [26]. Individuals in neighborhoods with low SES are likely to have small financial margins [27], which might affect their willingness to pay for prescribed drugs. The findings of the present study indicate that the lower odds of statin medication in MI patients living in deprived neighborhoods may be explained by individual-level socioeconomic factors. This could imply that the neighborhood in itself does not affect statin medication in this patient group.

Although the present study was unable to detect a neighborhood effect on statin medication in CHD patients, previous studies have shown that neighborhood-level deprivation is a strong predictor for CHD incidence and fatality from CHD after adjusting for individual-level sociodemographic characteristics [5]. In addition, a recent study of ours has shown that neighborhood-level deprivation affects prescription patterns of statins in patients with atrial fibrillation, after adjusting for individual-level factors [28]. Adults with atrial fibrillation living in high SES neighborhoods were more often prescribed statins (men and women, OR 1.23) compared to their counterparts residing in middle SES neighborhoods [28]. It is unclear why neighborhood-level deprivation affects statin medication for patients with atrial fibrillation, but not for the MI patients in the present study.

One possible explanation could be that statin medication after MI is initiated almost exclusively at the hospital, i.e., in immediate relation to the event, whereas statin medication, for other reasons, might be initiated at a local health care center where the potential neighborhood effects may be stronger than in hospitals that cover a wider geographic area. Previous research has shown that prescription patterns among doctors working in local health care centers may be affected by neighborhood SES [29]. Additionally, individuals living in more affluent neighborhoods may be better informed as patients, irrespective of individual-level SES.

Finally, differences in medication patterns may in some cases be explained by “medication deserts”, where pharmacies in low SES neighborhoods may have lower availability of drugs [30]. In Sweden, with universal health care and good availability of drugs, this is unlikely a problem.

The results of the current study, including all MI patients in Sweden during the study period, show that statin medication rates for these patients are generally high. Of the 116,840 patients with MI, 89.7 % received statins. For comparison, 89 and 88 % of patients were treated with statins according to Italian data from the REAL registry [31] and French data from the FAST-MI registry [32], respectively.

In the US, several studies of statin medication for CHD patients have shown medication rates of about 60-70 % [33–35]. However, those studies only included a sub-set of MI patients, and the authors of one of the studies that reported high medication rates explained their findings as a result of only including highly motivated medical practices [36]. It has previously been shown that in countries without universal health insurance, lack of individual health insurance is associated with lower likelihood of being treated with a statin [35, 36]. It is also possible that availability of generic statins will result in lower costs for the patients and improved compliance. The high statin medication rates in this study indicates that universal health care may improve statin medication for MI patients, which could be an argument in support of providing universal health care to the entire population.

### Limitations and strengths

Our study has some limitations. Residual confounding may exist as socioeconomic measures only represent proxies for individual-level status. However, as the lower odds of statin medication in those MI patients living in deprived neighborhoods no longer remained significant after the statistical adjustments, it is likely that our socioeconomic measures are relatively good proxies of individual-level socioeconomic status. Another limitation is that we did not account for mobility, i.e., move to a different neighborhood during the follow-up period. However, we have previously checked the mobility in this age group and found it to be low. In addition, the follow-up period was relatively short, which implies that most people likely remained in their neighborhood during the study period.

This study has also, however, several strengths. The large cohort, which included practically all patients diagnosed with MI in Sweden during the study period, increases the generalizability of our results as very little data is missing. Another strength is the use of personal identification numbers, which made it possible to link health care data to socioeconomic data as well as following individuals in different national registers. A further strength is the use of the Hospital Discharge Register, which has high validity, especially for cardiovascular disorders [11–13]. Finally, the use of multilevel modeling helped us to separate neighborhood-level and individual-level effects and allowed for consideration of both random and fixed effects.

## Conclusion

Neighborhood-level SES was modestly associated with statin medication rates in MI patients and this association was no longer significant after adjusting for individual-level sociodemographic factors. These findings raise important clinical and public health concerns, and indicate that individual-level approaches may be most important in health care policies regarding statin medication in MI patients.

## Highlight

- This is the first study to determine whether neighborhood socioeconomic status is associated with statin medication rates in myocardial infarction (MI) patients.
- Low neighborhood-level socioeconomic status was significantly associated with lower statin medication rates (odds ratio 0.80).
- After adjustment for individual-level socioeconomic characteristics and comorbidities, this association was no longer significant.
- Individual-level approaches may be most important in health care policies regarding statin medication in MI patients.

## Abbreviations

CHD, Coronary heart disease; ICC, intraclass correlation; ICD, International Classification of Diseases; MI, myocardial infarction; OR, Odds ratio; SES, socioeconomic status

## Additional file

Additional file 1: Table S1. Evaluating the interaction between neighborhood deprivation and individual-level characteristics for statin medication in myocardial infarction patients. (DOCX 31 kb)

## References

[1] Mozaffarian D, Benjamin EJ, Go AS, Arnett DK, Blaha MJ, Cushman M (2015). Heart disease and stroke statistics--2015 update: a report from the American Heart Association. Circulation.

[2] Lynch JW, Kaplan GA, Cohen RD, Tuomilehto J, Salonen JT (1996). Do cardiovascular risk factors explain the relation between socioeconomic status, risk of all-cause mortality, cardiovascular mortality, and acute myocardial infarction?. Am J Epidemiol.

[3] Taylor F, Huffman MD, Macedo AF, Moore TH, Burke M, Davey Smith G (2013). Statins for the primary prevention of cardiovascular disease. Cochrane Database Syst Rev.

[4] Group Scandinavian Simvastatin Survival Study (1994). Randomised trial of cholesterol lowering in 4444 patients with coronary heart disease: the Scandinavian Simvastatin Survival Study (4S). Lancet.

[5] Winkleby M, Sundquist K, Cubbin C (2007). Inequities in CHD incidence and case fatality by neighborhood deprivation. Am J Prev Med.

[6] Sundquist K, Winkleby M, Ahlen H, Johansson SE (2004). Neighborhood socioeconomic environment and incidence of coronary heart disease: a follow-up study of 25,319 women and men in Sweden. Am J Epidemiol.

[7] Cubbin C, Sundquist K, Ahlen H, Johansson SE, Winkleby MA, Sundquist J (2006). Neighborhood deprivation and cardiovascular disease risk factors: protective and harmful effects. Scand J Public Health.

[8] Sundquist K, Malmstrom M, Johansson SE (2004). Neighbourhood deprivation and incidence of coronary heart disease: a multilevel study of 2.6 million women and men in Sweden. J Epidemiol Community Health.

[9] Sundquist K, Li X (2006). Coronary heart disease risks in first- and second-generation immigrants in Sweden: a follow-up study. J Intern Med.

[10] Kawakami N, Winkleby M, Skog L, Szulkin R, Sundquist K (2011). Differences in neighborhood accessibility to health-related resources: a nationwide comparison between deprived and affluent neighborhoods in Sweden. Health Place.

[11] Ludvigsson JF, Andersson E, Ekbom A, Feychting M, Kim JL, Reuterwall C (2011). External review and validation of the Swedish national inpatient register. BMC Public Health.

[12] Ingelsson E, Arnlov J, Sundstrom J, Lind L (2005). The validity of a diagnosis of heart failure in a hospital discharge register. Eur J Heart Fail.

[13] Nilsson AC, Spetz CL, Carsjo K, Nightingale R, Smedby B (1994). Reliability of the hospital registry. The diagnostic data are better than their reputation. Lakartidningen.

[14] Zöller B, Li X, Sundquist J, Sundquist K (2012). Neighborhood deprivation and hospitalization for venous thromboembolism in Sweden. J Thromb Thrombolysis.

[15] Zöller B, Li X, Sundquist J, Sundquist K (2013). Neighbourhood deprivation and hospitalization for atrial fibrillation in Sweden. Europace.

[16] Li X, Sjöstedt C, Sundquist K, Zöller B, Sundquist J (2014). Neighborhood deprivation and childhood autism: a nationwide study from Sweden. J Psychiatr Res.

[17] Sundquist J, Johansson SE, Yang M, Sundquist K (2006). Low linking social capital as a predictor of coronary heart disease in Sweden: a cohort study of 2.8 million people. Soc Sci Med.

[18] Johnell K, Lindstrom M, Melander A, Sundquist J, Eriksson C, Merlo J (2006). Anxiolytic-hypnotic drug use associated with trust, social participation, and the miniaturization of community: a multilevel analysis. Soc Sci Med.

[19] Johnell K, Lindstrom M, Sundquist J, Eriksson C, Merlo J (2006). Individual characteristics, area social participation, and primary non-concordance with medication: a multilevel analysis. BMC Public Health.

[20] Sundquist K, Theobald H, Yang M, Li X, Johansson SE, Sundquist J (2006). Neighborhood violent crime and unemployment increase the risk of coronary heart disease: A multilevel study in an urban setting. Soc Sci Med.

[21] Snijders PTABB, Bosker TABSRJ, Bosker PRJ (1999). Multilevel analysis: an introduction to basic and advanced multilevel modeling: SAGE.

[22] Brooks JM, Cook EA, Chapman CG, Kulchaitanaroaj P, Chrischilles EA, Welch S (2014). Geographic variation in statin use for complex acute myocardial infarction patients: evidence of effective care?. Med Care.

[23] Reid FD, Cook DG, Whincup PH (2002). Use of statins in the secondary prevention of coronary heart disease: is treatment equitable?. Heart.

[24] Headen AE, Masia NA, Axelsen KJ (2006). Effects of medicaid access restrictions on statin utilisation for patients treated by physicians practising in poor and minority neighbourhoods. PharmacoEconomics.

[25] Tuppin P, Ricci-Renaud P, de Peretti C, Fagot-Campagna A, Alla F, Danchin N (2014). Frequency of cardiovascular diseases and risk factors treated in France according to social deprivation and residence in an overseas territory. Int J Cardiol.

[26] Thiebaud P, Patel BV, Nichol MB (2008). The demand for statin: the effect of copay on utilization and compliance. Health Econ.

[27] Carlsson A, Starrin B, Gigante B, Leander K, Hellenius M-L, de Faire U (2014). Financial stress in late adulthood and diverse risks of incident cardiovascular disease and all-cause mortality in women and men. BMC Public Health.

[28] Carlsson AC, Wändell P, Gasevic D, Sundquist J, Sundquist K (2015). Neighborhood deprivation and warfarin, aspirin and statin prescription - A cohort study of men and women treated for atrial fibrillation in Swedish primary care. Int J Cardiol.

[29] Pimenta E, Stowasser M (2009). Uncontrolled hypertension: beyond pharmacological treatment. Hypertens Res.

[30] Amstislavski P, Matthews A, Sheffield S, Maroko A, Weedon J (2012). Medication deserts: survey of neighborhood disparities in availability of prescription medications. Int J Health Geogr.

[31] Campo G, Guastaroba P, Marzocchi A, Santarelli A, Varani E, Vignali L (2013). Impact of COPD on long-term outcome after ST-segment elevation myocardial infarction receiving primary percutaneous coronary intervention. Chest.

[32] Hanssen M, Cottin Y, Khalife K, Hammer L, Goldstein P, Puymirat E (2012). French registry on acute ST-elevation and non ST-elevation myocardial infarction 2010. FAST-MI 2010. Heart.

[33] Johansen ME, Green LA, Sen A, Kircher S, Richardson CR (2014). Cardiovascular risk and statin use in the United States. Ann Fam Med.

[34] Goff DC, Gu L, Cantley LK, Sheedy DJ, Cohen SJ (2003). Quality of care for secondary prevention for patients with coronary heart disease: results of the Hastening the Effective Application of Research through Technology (HEART) trial. Am Heart J.

[35] Johansen ME, Hefner JL, Foraker RE (2015). Antiplatelet and statin use in US patients with coronary artery disease categorized by race/ethnicity and gender, 2003 to 2012. Am J Cardiol.

[36] Arnold SV, Spertus JA, Tang F, Krumholz HM, Borden WB, Farmer SA (2011). Statin use in outpatients with obstructive coronary artery disease. Circulation.

