# Supplementary material for: Neighborhood socioeconomic characteristics and statin medication in patients with myocardial infarction: a Swedish nationwide follow-up study
Source: BMC Cardiovasc Disord. 2016 Jul 8;16:146. doi: 10.1186/s12872-016-0319-y (PMC4938992; doi:10.1186/s12872-016-0319-y)
Supplement: Additional file 1: Table S1. — Evaluating the interaction between neighborhood deprivation and individual-level characteristics for statin medication in myocardial infarction patients. (DOCX 31 kb) [file 12872_2016_319_MOESM1_ESM.docx]

| **Supplementary Table S1. Evaluating the interaction between neighborhood deprivation and individual-level characteristics for statin medication in myocardial infarction patients.** | | | | | | | | | | | | |
| --- | --- | --- | --- | --- | --- | --- | --- | --- | --- | --- | --- | --- |
|  | Educational level | | | | | | | | | | | |
|  | ≤ 9 | | |  | 10-12 | | |  | > 12 | | | |
|  | OR | 95% CI | |  | OR | 95% CI | |  | OR | 95% CI | |  |
| Neighborhood-level SES |  |  |  |  |  |  |  |  |  |  |  |  |
| High | 0.82 | 0.73 | 0.91 |  | 1.04 | 0.91 | 1.18 |  | ref. | | | |
| Middle | 0.90 | 0.82 | 0.99 |  | 1.17 | 1.06 | 1.29 |  | 0.99 | 0.90 | 1.09 |  |
| Low | 0.89 | 0.80 | 0.98 |  | 1.05 | 0.93 | 1.19 |  | 0.94 | 0.81 | 1.08 |  |
| \| Adjusted for age, sex, family income, marital status, country of origin, urban/rural status. \|  \| \| --- \| --- \| | | | | | | | | | | | | |
